# Supplementary material for: MetaRibo-Seq measures translation in microbiomes
Source: Nat Commun. 2020 Jun 29;11:3268. doi: 10.1038/s41467-020-17081-z (PMC7324362; doi:10.1038/s41467-020-17081-z)
Supplement: Supplementary file 10 — Supplementary Data 7 [file 41467_2020_17081_MOESM10_ESM.zip › File2/Confidence_High_Taxonomy/221247_out.krona.html]

Javascript must be enabled to view this page.

members
magnitude
magnitudeUnassigned
count
unassigned
taxon
rank

221247\_out

7

2
superkingdom
7

7
phylum
1239

91061
7
class

order
7
186826

7
family
1300

1301
genus
7

671232
species group
1

76860
species
1

184250

SRS011243\_contig\_number\_contig-100\_18335.18335
1
subspecies

1304
species

SRS023352\_contig\_number\_43232SRS045715\_contig\_number\_41340SRS075404\_contig\_number\_12510SRS077633\_contig\_number\_contig-100\_2783.152116SRS142896\_contig\_number\_contig-100\_4927.181688
5

1318

SRS143529\_contig\_number\_67157
1
species
